# Cultivation of a *Synergistetes* strain representing a previously uncultivated lineage

**DOI:** 10.1111/j.1462-2920.2009.02135.x

**Published:** 2010-04

**Authors:** S R Vartoukian, R M Palmer, W G Wade

**Affiliations:** King's College London Dental Institute, Infection Research Group, Guy's CampusLondon SE1 9RT, UK

## Abstract

Subgingival plaque samples obtained from human subjects with periodontitis, shown to include previously uncultivable members of the phylum *Synergistetes*, were used to inoculate Cooked Meat Medium (CMM). The presence of Cluster A (uncultivable) *Synergistetes* was monitored by fluorescent *in situ* hybridization (FISH) and quantitative PCR (Q-PCR). Cluster A *Synergistetes* were found to grow in CMM in co-culture with other plaque bacteria and growth was stimulated by the addition of mucin and serum. Plaque samples were also used to inoculate Blood Agar (BA) plates and growth of Cluster A *Synergistetes* was revealed after anaerobic incubation, by colony hybridization with specific probes. Surface growth on the plates in regions identified by colony hybridization was harvested and used to inoculate fresh plates, thus enriching for Cluster A *Synergistetes*. Cross-streaks of other plaque bacteria were also used to stimulate *Synergistetes* growth. In the early passages, no discrete *Synergistetes* colonies were seen, but after eight passages and the use of cross-streaks of other bacteria present in the enriched community, colonies arose, which consisted solely of Cluster A *Synergistetes* cells, as determined by 16S rRNA gene PCR and cloning. This is the first report of the successful culture of a member of the uncultivable branch of this phylum.

## Introduction

Bacteria belonging to the recently described phylum *Synergistetes* (Jumas-Bilak *et al.*, 2009) are widespread in the environment and in animals (Godon *et al.*, 2005). As yet, the vast majority of the 16S rRNA sequences that are available from the phylum have been obtained from culture-independent studies, but 40 strains from 11 genera have been isolated (Hugenholtz *et al.*, 2009). In humans, *Synergistetes* have frequently been detected at sites of disease: soft tissue wounds, cysts and abscesses; blood; peritoneal fluid; and the oral cavity in subjects with dental disease (Munson *et al.*, 2002; Horz *et al.*, 2006; de Lillo *et al.*, 2006; Bernard *et al.*, 2007; Jumas-Bilak *et al.*, 2007; Downes *et al.*, 2009).

**Fig. 1:**
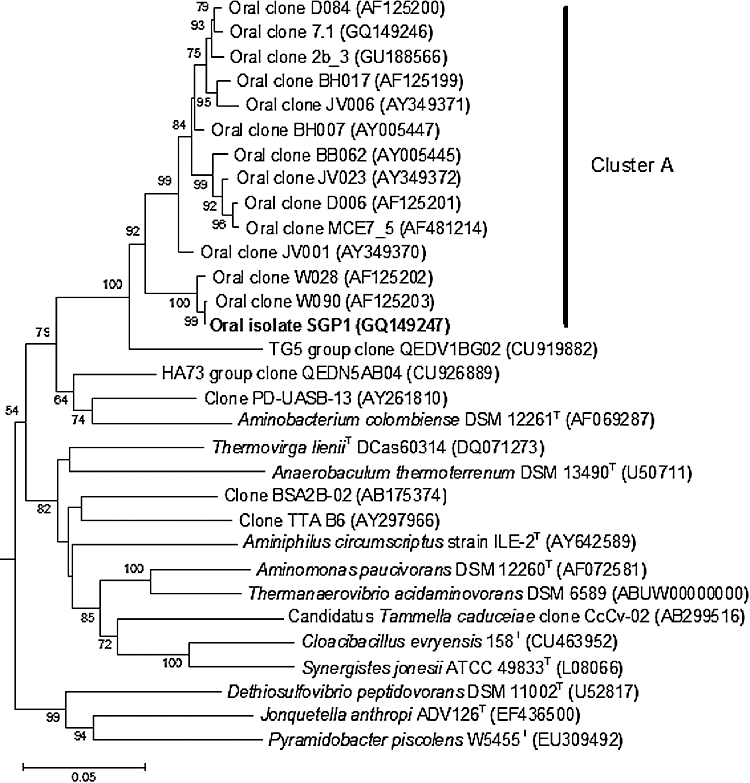
Phylogenetic tree based on 16S rRNA gene sequence comparisons over 1221 aligned bases showing relationship between *Synergistetes* strain SGP1, Cluster A *Synergistetes* and representatives of other genera and groups within the phylum *Synergistetes*. Tree was constructed using the neighbour-joining method following distance analysis of aligned sequences. Numbers represent bootstrap values for each branch based on data for 500 trees. Accession numbers for 16S rRNA sequences are given for each strain. Scale bar shows number of nucleotide substitutions per site.

Oral *Synergistetes* taxa fall into two main Clusters, A and B (Vartoukian *et al.*, 2007). The *Synergistetes* Cluster A comprises 22 taxa, related to the TG5 group (Hugenholtz *et al.*, 2009) (Fig. 1). These taxa are exclusively oral but none have ever been cultivated in the laboratory, despite their widespread prevalence when molecular methods are used for their detection (Vartoukian *et al.*, 2009). It is estimated that half of the approximately 700 taxa found in the human oral cavity have yet to be cultured (Paster *et al.*, 2006). The study of such bacteria is clearly important in order to improve our understanding of this complex environment, its bacterial inhabitants, their interactions and their potential role in disease. Bacteria must be cultivated in purity before they can be fully characterized not only with regard to their phenotypic and physiological properties, but also their virulence potential and susceptibility to antimicrobials.

Bacteria that are unable to grow *in vitro* may be dependent on specific factors or signals present only within the source habitat (Lewis, 2007). The provision of such factors, for example by artificially simulating the natural environment (Kaeberlein *et al.*, 2002; Bollmann *et al.*, 2007; Ferrari *et al.*, 2008), may encourage growth. These essential factors may include specific nutrients not available in conventional culture media or chemical signals from other bacteria within the local community (Davey, 2008). Dental plaque is an example of a multi-species biofilm, within which a number of interspecies interactions and co-dependent relationships have been observed (Marsh, 2005; Kuramitsu *et al.*, 2007). An example of this is the metabolic interaction between *Tannerella forsythia* and *Fusobacterium nucleatum*– production of N-acetyl muramic acid (NAM) by *F. nucleatum* supports the growth of *T. forsythia*, which is unable to synthesize NAM, an essential factor in cell wall synthesis (Wyss, 1989).

Based on the hypothesis that Cluster A *Synergistetes* have not been cultured because of their dependence on other dental plaque bacteria for growth, the aims of this work were: (i) to investigate whether as-yet-uncultivated Cluster A *Synergistetes* are able to grow in co-culture *in vitro* with dental plaque bacteria in a complex broth medium, and (ii) to attempt to isolate members of the Cluster A *Synergistetes* in pure culture on solid media, using colony hybridization as a guide to enrichment.

## Results

### Quantitative PCR assay development

A Quantitative PCR (Q-PCR) assay for Cluster A *Synergistetes* was developed. Eight candidate primer sets were found to give linear data over a range of at least five of the nine calibration standards in dilution series. Of the primers targeting *Synergistetes*, 763F/827R demonstrated the broadest range of linearity (10^−1^–10^−8^), the highest linear regression coefficient (R^2^ value of 0.999), a large cycle threshold (C_t_) value of 33 for the no-template control and a distinct melting curve profile confirming reaction specificity. Sequence analysis of clone libraries prepared from the pooled products of two replicate end-point PCR reactions with primers 763F/827R, mixed DNA template A6G and an annealing temperature of 57°C showed that all 42 clones sequenced were members of the Cluster A *Synergistetes* confirming the validity of the primers. A dilution series of genomic DNA was prepared from a mixed template sample taken from a Cooked Meat Medium (CMM) culture, and Q-PCR assay was performed with primer sets 763F/827R and 1406F/1492R. Both primer sets showed good proportional amplification and linearity with R^2^ values between 0.997 and 0.999.

### Growth of cluster A *Synergistetes* in co-culture in Cooked Meat Medium

Fluorescent *in situ* hybridization (FISH) analysis of the plaque samples collected from subjects F5 and F6 confirmed the presence of Cluster A *Synergistetes*, including *Synergistetes* OTU 3.3. Of the CMM broths and phosphate-buffered saline (PBS) controls inoculated with these samples, Cluster A *Synergistetes* were detected after 5 and 10 days anaerobic incubation in the CMM only. In the original plaque samples, *Synergistetes* cells were curved bacilli 2–4 µm in length. At 5 days of culture, some cells were elongated and 6–7 µm long. By 10 days, the majority of the cells were filamentous, up to 13 µm in length. Subculture of the primary CMM cultures to fresh CMM at 2, 5 and 10 days revealed, in each case, Cluster A *Synergistetes* by FISH after 5 days of incubation. No *Synergistetes* were detected in subcultures to PBS at the same time points. All positive CMM sub-cultures contained only small numbers of *Synergistetes* cells with a maximum of five fluorescent cells in each field of view equivalent to a count of 4.5 × 10^4^ cells per ml of CMM broth.

**Fig. 2:**
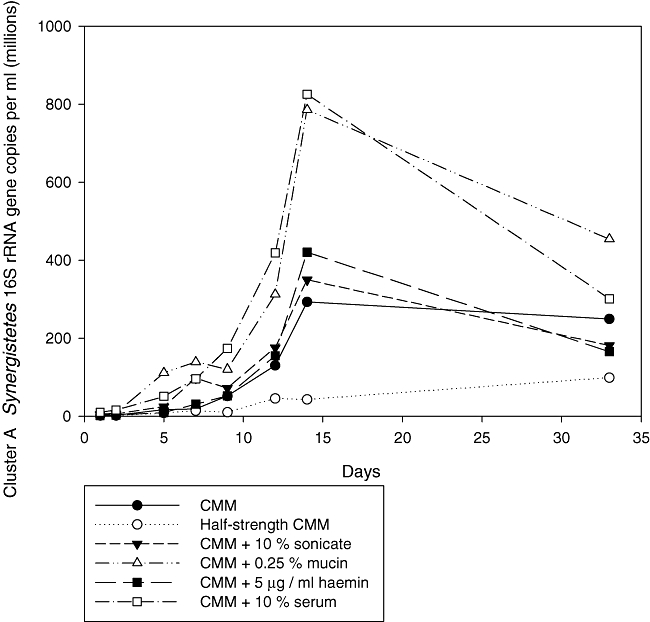
Cluster A *Synergistetes* 16S rRNA gene copies in CMM broths inoculated with a mixed culture of sub-gingival plaque.

Plain CMM and modified or supplemented CMM were inoculated with cells harvested from Blood Agar (BA) plate cultures enriched for Cluster A *Synergistetes*. The results of Q-PCR determination of Cluster A *Synergistetes* 16S rRNA gene counts within the broth cultures are shown in Fig. 2. For all media except half-strength CMM, counts showed a marked increase until 14 days of growth, followed by a partial decrease at 33 days. In contrast, the half-strength CMM showed only a modest increase, which continued until day 33. The corresponding 16S rRNA gene copy numbers for total bacteria (around 10^10^ gene copies per ml of broth) increased up to day 14 and declined moderately thereafter. The doubling time for Cluster A *Synergistetes* was determined as 50.4 h for CMM/serum during log phase. The highest counts of Cluster A *Synergistetes* were found in CMM supplemented with serum or mucin, while the half-strength CMM had the lowest (Fig. 2).

**Fig. 3:**
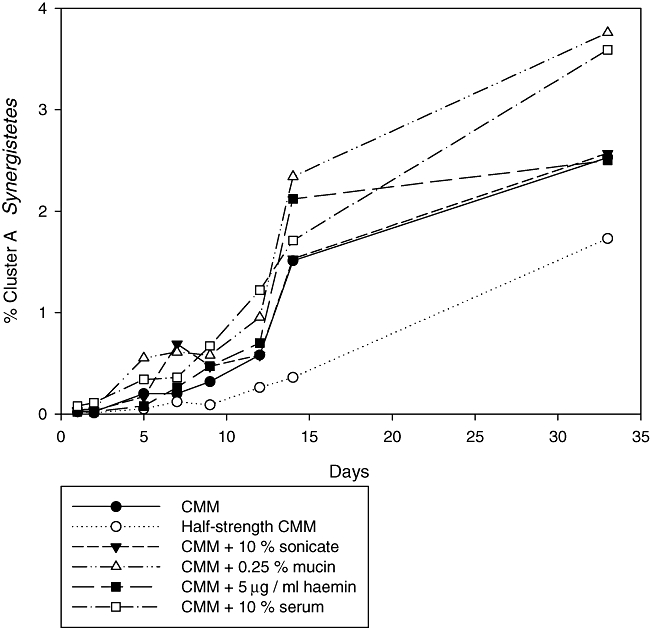
Proportion of Cluster A *Synergistetes* within the total bacteria in CMM broths inoculated with a mixed culture of sub-gingival plaque.

**Fig. 4:**
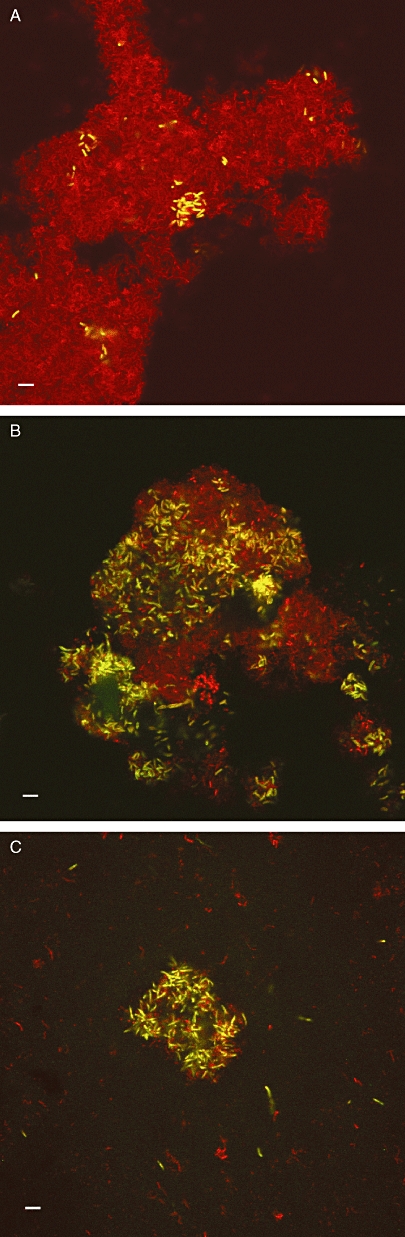
Cluster A *Synergistetes* in CMM/serum broth visualized by FISH with probe A_487, Cy3 (yellow) and total bacteria with probe EUB338, Cy5 (red) at days: (A) 1, (B) 14 and (C) 33 of growth. Bars, 10 µm.

In all broths, the proportion of Cluster A *Synergistetes* to total bacterial count increased over time (Fig. 3). Cluster A *Synergistetes* were particularly enriched in CMM/serum and CMM/mucin, making up 3.6% and 3.7%, respectively, of the total bacterial gene counts at 33 days (Fig. 3). FISH analysis confirmed the presence of Cluster A *Synergistetes* in the CMM broths at all time points. Furthermore, the FISH images showed temporal changes in the abundance of Cluster A *Synergistetes* and relative increases in the proportion of these bacteria within the total over the course of the experiment (Fig. 4), which mirrored the patterns observed in the Q-PCR data. *Synergistetes* cells formed large clumps and aggregates with other bacteria in the mucin- and serum-supplemented media (Fig. 4).

### Colony hybridization-directed isolation of Cluster A *Synergistetes* in pure culture

Isolation of Cluster A *Synergistetes* strains was attempted from FISH-positive samples collected from subjects F7 and F9. Colony hybridization of BA plates inoculated with sample F7 revealed colocalization of positive detections with probes A_487, A_845 and 3.3_65, but no colonies subcultured from these regions were identified as *Synergistetes* on purification. 16S rRNA gene sequence analysis identified isolates growing at the hybridization detection regions as *Prevotella baroniae*, *Peptostreptococcus stomatis*, *Parvimonas micra*, *Dialister pneumosintes* or *Campylobacter rectus*.

**Fig. 5:**
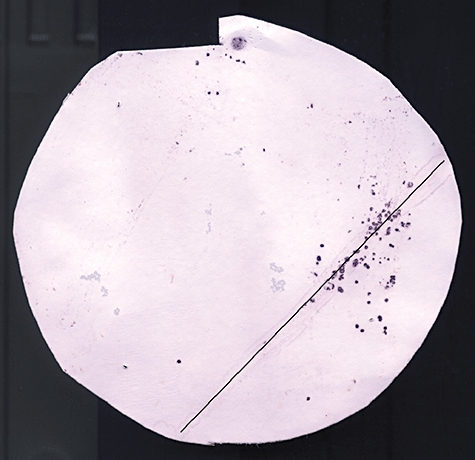
Blot from streak plate G hybridized at day 14 with probe 3.3_65; position of *S. aureus* streak indicated by line.

**Fig. 6:**
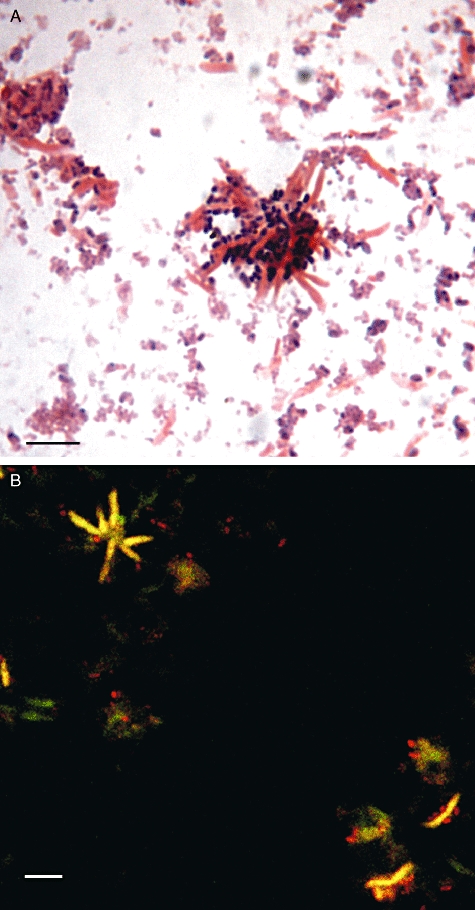
Cells harvested from a colony hybridization-positive site on streak plate G at day 15 of culture: (A) Gram stain and (B) FISH analysis of same slide after Gram-staining; overlay of Cluster A *Synergistetes* in yellow (probe A_487, Cy3) with total bacteria in red (probe EUB338, Cy5). Bars, 10 µm.

For sample F9, initial positive colony hybridization detections were additionally tested by FISH and it was confirmed that Cluster A *Synergistetes* cells were present at sites of hybridization detection. For example, of 10 colony hybridization regions positive for probe 3.3_65, nine were positive by FISH with probe A_487. Serial subculture of cells from positive hybridization regions of the plate allowed the establishment of a stable plate culture of Cluster A *Synergistetes*, although individual pure colonies were not formed. The mixed community thus established on BA plates in anaerobic culture after five passages was identified by 16S rRNA gene sequence analysis. Isolates of four species were identified: *P. micra*, *C. rectus*, *T. forsythia* and *Anaeroglobus geminatus*. Sequencing of clone libraries prepared from the cultures revealed the same four species and *Synergistetes* Oral Taxon 363 (Human Oral Microbiome Database designation), which includes phylotype W090, a member of Cluster A. In order to encourage independent growth of the target organism, cross-streaks of candidate donor organisms were inoculated onto some passage plates. The colony hybridization reactions of streak plate G (passage 5) revealed that detections of Cluster A *Synergistetes* were most numerous around the *Staphylococcus aureus* streak (Fig. 5), although no discrete *Synergistetes* colonies were found. However, microscopic examination of Gram-stained smears from the regions of hybridization detections revealed the presence of large Gram-negative curved bacilli in close proximity and apparently coaggregating with small Gram-positive cocci, resembling *P. micra* (Fig. 6A). The FISH analysis was performed on the same Gram-stained slide, and confirmed the identity of the large cells as Cluster A *Synergistetes* (Fig. 6B). Interestingly, these bacteria were arranged in rosette formations.

A CMM/serum broth was inoculated with a hybridization-positive region of cells from plate G, and the culture was examined by dark-field microscopy at 7 and 14 days of incubation. Bacilli, of the same size and shape as the Cluster A *Synergistetes* seen in the Gram-stained smears from plate G, were found to be actively motile at both 7 and 14 days. Formation of rosettes was again observed within the older 14-day culture.

**Fig. 7:**
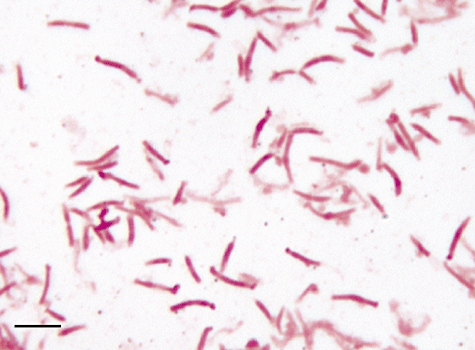
Gram-stained cells from an isolated 16-day colony of *Synergistetes* SGP1. Bar, 10 µm.

**Fig. 8:**
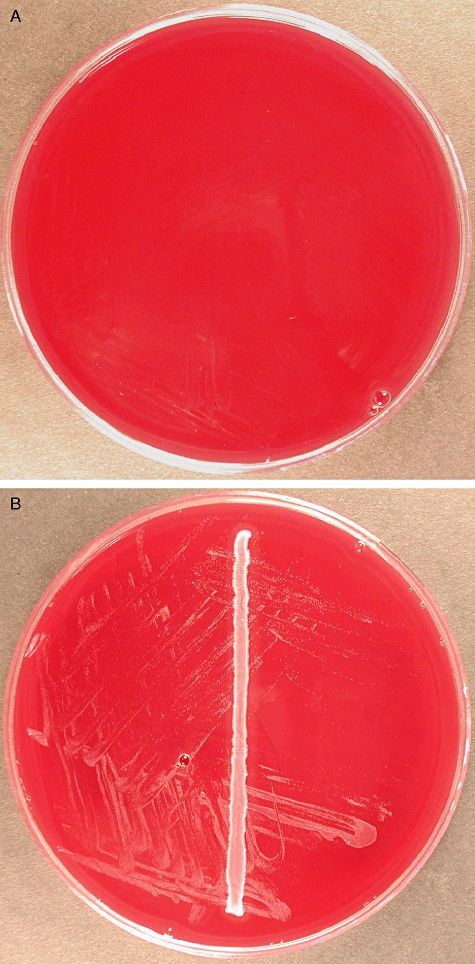
Ten-day cultures of *Synergistetes* strain SGP1: (A) No donor streak, (B) *P. micra* donor.

At passage 8, two streak plates (CH and CG) were prepared with inocula from regions of cells corresponding to hybridization detections and cross-streaked with all four of the strains that were present within the mixed cultures with *Synergistetes*: *P. micra*, *C. rectus*, *T. forsythia* and *A. geminatus*. Blots from these plates showed multiple colony hybridization detections which, for the first time, were found to correspond to discrete colonies, all of which had a similar morphology and did not resemble the colonies of any of the four species previously isolated. Gram-staining (Fig. 7) and FISH analysis of hybridization-targeted colonies provisionally identified them as Cluster A *Synergistetes* and this was confirmed by 16S rRNA gene sequence analysis, which identified the organism as *Synergistetes* oral taxon 363. The strain was designated *Synergistetes* SGP1 (Fig. 1). Initial sub-culture of *Synergistetes* SGP1 showed that independent growth was extremely slow with only microcolonies 0.01 mm in diameter visible on BA plates after 16 days anaerobic incubation. After three subcultures over 5 weeks, growth improved with, in some cases, colonies up to 0.3 mm in diameter seen after 10 days of incubation. In addition, the organism developed four distinct colony types on successive subculture. In case these represented mixed cultures, 16S rRNA gene clone libraries were prepared from each type and 16 clones sequenced. All 64 clones were identified as *Synergistetes* Oral Taxon 363. Although independent growth of *Synergistetes* SGP1 remained indifferent, cross-streaks of *F. nucleatum*, *P. micra*, *S. aureus*, and to a lesser degree, *T. forsythia,* markedly stimulated its growth with the formation of larger and faster-growing colonies. Figure 8 shows minimal growth in the absence of a donor organism, and stimulation by *P. micra*. *C. rectus*, *A. geminatus* and *P. baroniae* did not stimulate growth.

Enzyme profiles for *Synergistetes* SGP1 (cultured independently of helper strains) were assessed in triplicate using the Rapid ID 32A anaerobe identification kit (bioMerieux) and revealed strong positive results for leucyl glycine arylamidase and glycine arylamidase alone, giving an enzyme profile of 0000 0404 00.

**Fig. 9:**
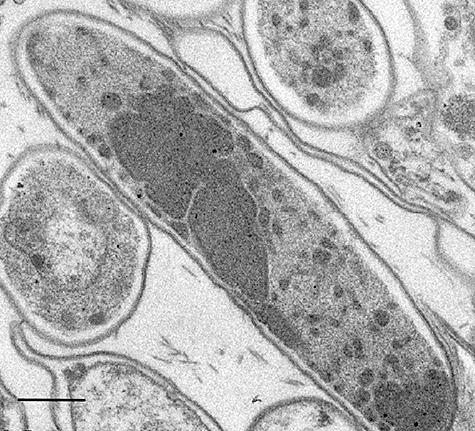
Transmission electron micrograph of an ultrathin section showing cells of *Synergistetes* SGP1 harvested from 10-day colonies cultured in the presence of *F. nucleatum*. Bar, 0.5 µm.

Ultrastructural analysis using transmission electron microscopy as described previously (Downes *et al.*, 2002) showed a distinctive internal structure comprising microcompartment organelles (Fig. 9), which appeared more numerous within cells cultured in the presence of *F. nucleatum* than in those growing independently.

A summary of the established morphological and physiological features of *Synergistetes* SGP1 includes: motile, Gram-negative, size-variable curved bacilli with a tendency to cluster into rosettes; variable colony size and morphology; obligately anaerobic growth, weak in the absence of specific helper strains; presence of intracellular microcompartments; and strong aminopeptidase activity.

## Discussion

Using a combination of molecular and cultural analyses, the work described here has resulted in the cultivation in co-culture of the previously uncultivated Cluster A *Synergistetes* and the ultimate isolation of *Synergistetes* SGP1, the first member of this group to have been purified.

On the assumption that Cluster A *Synergistetes* are proteolytic obligate anaerobes (as are all *Synergistetes* species that have been characterized to date), the initial investigation of the growth of these bacteria within co-cultures was undertaken in CMM (a complex proteinaceous medium that is particularly suited for the growth and enrichment of proteolytic anaerobes) and tested by FISH. Since the FISH method targets rRNA, which is essential to basic cellular metabolism and is thought to degrade soon after cell death (Nocker and Camper, 2009), the results, which revealed strong positive FISH signals for Cluster A *Synergistetes* in the plaque-inoculated CMM at 5 and 10 days of incubation but negative results for the inoculated control media at equivalent time points, indicate that the Cluster A *Synergistetes* were able to grow in the cooked meat mixed cultures.

It was noted, however, that although Cluster A *Synergistetes* were successfully passaged at 10 days into fresh CMM, actual numbers of fluorescent cells per field of view were low, signifying that these bacteria were not thriving under these conditions. Furthermore, their cellular morphology changed from smaller curved bacilli in the baseline plaque sample and at 2 days of growth in the CMM, into more elongated forms (including filaments) at later stages of growth (5 and 10 days), providing further evidence that these bacteria were growing suboptimally and under environmental stress. The induction of morphological change in cells that are subjected to marginal growth conditions is not uncommon. Harborth and Hanert (1982) determined that cells of *Selenomonas ruminantium* became crescent-shaped and sinuous in unfavourable growth conditions, when growth was slow. Environmental conditions that have been known to induce morphological change in bacteria include the presence of oxygen (Eisenberg, 1973), low humidity levels (de Goffau *et al.*, 2009) and extremes of pH or temperature (Mattick *et al.*, 2003a); and it has been suggested that the filamentation of *Salmonella* cells under suboptimal growth conditions may be the result of an early block in cell septation (Mattick *et al.*, 2003b).

Following on from the original experiment, a quantitative exploration of growth in modified or supplemented CMMs was undertaken, with a view to finding a more favourable medium for Cluster A *Synergistetes*. In contrast to the earlier finding that *Synergistetes* cells appeared to be struggling in the plain CMM cultures, an exponential rise was seen in Cluster A *Synergistetes* gene counts up to 14 days, suggestive of good growth. Furthermore, FISH analysis revealed that Cluster A *Synergistetes* cells were brightly staining even at 33 days of incubation, at which point other bacteria, hybridized with the universal probe EUB338, generally appeared fainter. Since the inoculum for this experiment was prepared from a BA plate culture that was enriched for *Synergistetes* following repeated passage guided by colony hybridization, this suggests that growth of Cluster A *Synergistetes* in the CMM was facilitated either by a reduction in bacterial competition or by a period of prior adjustment to *in vitro* conditions (through repeated subculture on solid media). The adaptation of bacteria to selective environmental pressures resulting in the development of ‘domesticated’ variants of previously unculturable bacteria after repeated passage with helper strains has been demonstrated for a *Psychrobacter* sp. (Nichols *et al.*, 2008).

Cluster A *Synergistetes* were enriched over the course of the experiment, indicating that the bacteria were adapting to growth under the broth conditions provided. Even so, doubling times for Cluster A *Synergistetes* in the mixed cultures were long – 50.4 h for the CMM with serum. These bacteria appear to be slow-growing compared with others such as the oral anaerobe *F. nucleatum*, which has a reported doubling time of 3.5 h (Rogers *et al.*, 1991) and the environmental *Synergistetes* strain *Aminomonas paucivorans*, with a generation time of 16 h under optimal conditions (Baena *et al.*, 1999).

Cooked Meat Medium supplemented with cell sonicate showed only a slight enhancement of growth, perhaps because the potential growth-supporting factors within the cell-free extract were labile (Rhee *et al.*, 2000; Kim *et al.*, 2008) or because the mixed bacterial suspension used to inoculate the broths already included donor strains, giving the medium supplemented with sonicate no additional advantage over the plain CMM. The mucin- and serum-supplemented media appeared to be the most favourable for the growth of Cluster A *Synergistetes*. These bacteria may possess glycosidic or proteolytic ability, suited to the catabolism of the specific glycoprotein and protein substrates in mucin and serum (Beighton *et al.*, 1988; Glenister *et al.*, 1988; ter Steeg *et al.*, 1988; Macfarlane *et al.*, 1989; Homer *et al.*, 1994). Cooperation of multiple plaque bacteria including the *Synergistetes* may have been responsible, through enzyme complementation, for the degradation of these complex substrates. Such metabolic cooperation has been reported previously for the plaque-bacteria-mediated catabolism of mucin glycoproteins (Bradshaw *et al.*, 1994; Wickstrom and Svensater, 2008) and serum components (ter Steeg *et al.*, 1988), including the main protein, albumin (Homer and Beighton, 1992; Wei *et al.*, 1999). In view of the amino acid degrading capabilities of all of the *Synergistetes* species that have been characterized to date, it is proposed that the Cluster A bacteria may likewise possess aminopeptidase activity and have a role in synergistic proteolytic degradation with cooperating bacteria. In the case of *Synergistetes* SGP1, a member of Cluster A, testing for pre-formed enzymes, confirmed positive results for certain aminopeptidases. Furthermore, the presence of intracellular microcompartments (another feature that appears to be characteristic of the phylum) may be of relevance here, as such organelles have been shown to comprise clusters of enzymes, which are involved in sequential metabolic activity (Cheng *et al.*, 2008).

The final part of this work demonstrated the colony hybridization-directed serial passage of Cluster A *Synergistetes* in mixed culture, which led ultimately to the isolation of *Synergistetes* SGP1. It was found that Cluster A *Synergistetes* were: (i) detectable on plate cultures by both FISH and colony hybridization, and (ii) amenable to passage (indicating cell viability). However until passage 8, these bacteria did not readily form visible colonies even after 15 days of incubation.

From the outset, it was suspected that Cluster A *Synergistetes* were dependent for growth on other bacteria within the mixed cultures, since: (i) they were detectable only in denser spread plate cultures or streak plate cultures where bacteria were in close proximity; (ii) none of the discrete colonies matching hybridization detections were identified as *Synergistetes*, hence the possibility that *Synergistetes* cells were growing within colonies of other bacterial species; and (iii) the blots with the greatest density of positive hybridization detections were derived from plates with donor cross-streaks. This suspicion was ultimately confirmed once *Synergistetes* strain SGP1 was purified and there was found to be enhanced growth in the presence of *P. micra* and *T. forsythia*– two of the bacteria that had been found in the original mixed cultures with *Synergistetes*. Other growth-stimulating species were *F. nucleatum* and *S. aureus*.

Corresponding phylotypes of the isolated strain *Synergistetes* SGP1 (*Synergistetes* W090/OTU 6.2) have been detected on several occasions using molecular methods (Paster *et al.*, 2001; Kumar *et al.*, 2005; 2006; Saito *et al.*, 2006; Vartoukian *et al.*, 2009). Phylotype *Synergistetes* W090 has been found associated with chronic periodontitis (Paster *et al.*, 2001; Kumar *et al.*, 2005); however, Kumar and colleagues (2003) reported conflicting results and a low prevalence ratio of 0.8 for *Synergistetes* W090 in disease. Furthermore, some recent work (Vartoukian *et al.*, 2009) found *Synergistetes* W090/OTU 6.2 to be prevalent and ‘abundant’ in both periodontally healthy subjects and those with chronic periodontitis, which would suggest that this bacterium is a commensal, endogenous to the oral cavity.

With the isolation of *Synergistetes* strain SGP1, a fastidious organism apparently dependent on a biofilm community lifestyle, as demonstrated by enhanced growth in the presence of certain other bacteria, comes the opportunity to develop our understanding of this bacterium and subsequently to apply this knowledge to the study of other as-yet-uncultivated organisms. With that in mind, future work will focus on characterizing this species comprehensively, as well as investigating the cooperative interactions between ‘feeder’ and *Synergistetes* strains. Furthermore, genomic analysis for the presence or absence of specific genes could provide invaluable information on possible metabolic interdependencies. A comparison of the genome of *Synergistetes* strain SGP1 with those of the readily cultivated cluster B *Synergistetes* species *P. piscolens* and *J. anthropi* may provide additional insight and enable the prediction of genes that are important in cultivability.

## Experimental procedures

### Subjects and samples

Four subjects with chronic periodontitis (aged 34–51 years, three male), participated in the study with their informed consent. Ethical approval was granted by Lewisham Research Ethics Committee (reference number 06/Q0701/35). Subgingival plaque samples were collected with a sterile curette from four deep periodontal pockets of 6–7 mm in each subject, pooled and suspended in sterile Reduced Transport Medium (Bowden and Hardie, 1971).

### Fluorescent *in situ* hybridization

The FISH was performed as described previously (Vartoukian *et al.*, 2009). The probes used were 3.3_65 (Alexa Fluor 488) targeting 16S rRNA of *Synergistetes* OTU 3.3 and A_487 (Cy3), specific for Cluster A *Synergistetes* (Vartoukian *et al.*, 2009), in combination with the universal bacterial probe EUB338 (Cy5) (Amann *et al.*, 1990). The nonsense probe NON338 (reverse sequence of EUB338) was used as a control.

### Quantitative PCR assay

16S rRNA gene Q-PCR primers were designed either to be ‘universal’ for the domain *Bacteria* or to target members of the Cluster A *Synergistetes* (Table 1). The *Synergistetes* primers were designed using the Primer 3 programme (http://frodo.wi.mit.edu), and modified where necessary, following visual inspection for sensitivity and specificity against a 16S rRNA gene sequence alignment, including cultivable *Synergistetes* species, oral *Synergistetes* phylotypes, a representative species from each genus found in the human oral cavity and members of the phylum *Deferribacteres*. Primer pairs were designed to produce an amplicon of 75–125 bp, with no regions of self-complementarity and with melting temperatures within 2°C of each other. Primer specificity was checked *in silico* by interrogation of the Ribosomal Database Project-II 16S rRNA database (Maidak *et al.*, 2001) and blast search of the GenBank nucleotide database.

**Table 1:** Primers for quantitative PCR.

Primers	Sequence (5′–3′)	Product size (bp)	16S rRNA gene target
673F	GAGTACCGGAGAGGCAAGTG	98	Cluster A *Synergistetes* (except W090, W028 & MCE3_120)
733R	AGTTACCGTCCAGCAAGTCG		
751F	CGACTTGCTGGACGGTAACT	115	Cluster A *Synergistetes* (except W090, W028 & MCE3_120)
827R	CATCTCTGCTCGCACACCTA		
751F	CGACTTGCTGGACGGTAACT	106	Cluster A *Synergistetes* (except W090, W028 & MCE3_120)
818R	TCGCACACCTAGCATTCATC		
760F	ACGGTAACTGACGCTGAG	95	Cluster A *Synergistetes* (except W090, W028 & MCE3_120)
818R	TCGCACACCTAGCATTCATC		
760F	ACGGTAACTGACGCTGAG	104	Cluster A *Synergistetes* (except W090, W028 & MCE3_120)
827R	CATCTCTGCTCGCACACCTA		
763F	GTAACTGACGCTGAGGTG	92	Cluster A *Synergistetes*
818R	TCGCACACCTAGCATTCATC		
763F	GTAACTGACGCTGAGGTG	101	Cluster A *Synergistetes*
827R	CATCTCTGCTCGCACACCTA		
1406F^a^	TTGYACACACCGCCCGT	124	‘Universal’ for bacteria
1492R^b^	TACGGYTACCTTGTTACGACTT		

a Single base modification to ‘universal’ primer by Lane (1991).

b Lane (1991).

The primer sets, synthesized by MWG_Biotech AG, were evaluated by PCR with DNA from plaque sample A6G, previously shown to contain Cluster A *Synergistetes* (Vartoukian *et al.*, 2009). PCR was performed using Reddy Mix PCR Master Mix (ABgene UK) with 5 pmol of each primer. Initial denaturation was performed at 95°C for 5 min, followed by 30 cycles of denaturation at 95°C for 45 s, annealing for 45 s at 57°C and extension at 72°C for 90 s, and a final 15 min extension at 72°C.

Positive control DNA for the quantification of Cluster A *Synergistetes* was generated by PCR amplification of a fragment of the 16S rRNA gene of phylotype *Synergistetes* OTU 2 with primers 94F (5′-GTGGTAACACGGAATGGT-3′) and ‘universal’ bacterial primer 1492R (Lane, 1991), and DNA from plaque samples A6F and A7G (known to include the taxon *Synergistetes* OTU 2). The amplification protocol was as described above but at an annealing temperature of 54°C. Clone libraries were prepared from the amplicons as described previously (Vartoukian *et al.*, 2009) and following standard M13 amplification, clone inserts were partially sequenced with primer 519R. Two clones identified as belonging to *Synergistetes* OTU 2 (*Synergistetes* OTU 2 clone 7.1 and *Synergistetes* OTU 2 clone 7.4) were fully sequenced. The GenBank accession numbers for these two sequences are GQ149246 and GQ149245 respectively.

PCR product from *Synergistetes* OTU 2 clone 7.1 was purified with the QIAquick PCR Purification Kit (Qiagen) following the manufacturer's instructions and then quantified using the NanoDrop 1000 spectrophotometer (Thermo Scientific) at 260 nm wavelength and 0.2 mm path length.

Calibration standards were prepared by 10-fold serial dilution of the purified PCR product to 10^−8^. Q-PCR was performed in a Rotagene 6000 real-time analyser (Corbett) with a total reaction volume of 10 µl, including 5 µl of SensiMix dT (SensiMix dT kit, Quantace), 0.2 µl of 50× SYBR Green 1 solution (SensiMix dT kit, Quantace), 2 µl of DNA template and 2 pmol each of forward and reverse primers (Table 1). The amplification protocol included initial denaturation at 95°C for 10 min, followed by 40 cycles of 95°C for 15 s, 57°C for 30 s and 72°C for 20 s. A final melting curve analysis was undertaken to evaluate reaction specificity.

The Q-PCR assay was ultimately used to quantify 16S rRNA genes for Cluster A *Synergistetes* and total bacteria in DNA extracted from six duplicate CMM broth types (see later). Each DNA sample was assayed in duplicate using the primers 763F/827R (for Cluster A *Synergistetes*) and 1406F/1492R (for total bacteria), and quantified by comparison against the dilution series of calibration standards (prepared from *Synergistetes* OTU 2 clone 7.1), which had a known number of 16S rRNA gene copies. The automated default algorithm determined the C_t_ value, which was converted to 16S rRNA gene copy number relative to the calibration standards and used to calculate the number of gene copies per ml of CMM broth. For each broth type at each time point, a mean figure was derived from four separate readings – two from the duplicate assays for each DNA sample giving a mean value, which was compared with the equivalent mean for the duplicate broth of the same type. In addition, gene counts of Cluster A *Synergistetes* were expressed as a proportion of the total bacterial gene counts by calculating the relevant percentage for each broth and then taking a mean of the equivalent values for duplicate broths of the same type.

### 16S rRNA gene sequencing

The 16S rRNA gene of strain *Synergistetes* SGP1 was sequenced as described previously (Vartoukian *et al.*, 2009). The sequence was deposited in the GenBank nucleotide sequence database with the Accession No. GQ149247.

### Growth of Cluster A *Synergistetes* in co-culture in Cooked Meat Medium

Twenty millilitres each of CMM (Oxoid, Poole, UK) and PBS medium (control) were inoculated with the plaque samples F5 and F6, and tested for the presence of Cluster A *Synergistetes* by FISH at 5 and 10 days of anaerobic incubation at 37°C. Cultures were passaged by transferring 250 µl of culture collected from the base of the meat layer to fresh medium. In a second experiment, a range of modified CMM broths were tested, including half-strength CMM, CMM + 0.25% type III mucin from porcine stomach, CMM + 5 µg ml^−1^ haemin and CMM + 10% donor horse serum. In addition, all colonies from a BA mixed culture plate derived from sample F9 and enriched for Cluster A *Synergistetes* by colony hybridization as described later, were harvested and suspended in 5 ml PBS. The suspension was sonicated for three pulses of 2 min, and, after removal of cell debris by centrifugation, the supernatant was passed through a 0.2 µm filter and stored at −70°C. Eighteen millilitres of CMM was supplemented with 2 ml of this sonicate. Duplicate broths of each type and a plain CMM were inoculated with 250 µl aliquots of a bacterial suspension prepared from a mixed culture of subgingival plaque enriched for Cluster A *Synergistetes* growing on BA Base no. 2 media (Laboratory M). Some 500 µl samples, harvested from immediately above the meat pellets, were collected from each broth after 1, 2, 5, 7, 9, 12, 14 and 33 days of anaerobic incubation. FISH analysis on 10 µl aliquots of neat broth samples was undertaken as described previously for the observation of target and total bacterial cell numbers over time. DNA was extracted from the remainder of each sample by means of the GenElute Bacterial Genomic DNA Kit (Sigma-Aldrich) according to the manufacturer's instructions, and assayed by Q-PCR.

### Colony hybridization-directed isolation of Cluster A *Synergistetes* in pure culture

Colonies were lifted from plates with a sterile 80 mm diameter nylon membrane (Amersham Hybond, GE Healthcare). One microlitre of DIG-labelled pBR328 (DIG Nucleic Acid Detection Kit, Roche Diagnostics) at a final concentration of 50 pg µl^−1^ and 1 µl of heat-denatured (95°C, 5 min) amplicon prepared by M13-amplification of the *Synergistetes* OTU 3.3 clone A3G_46 (Vartoukian *et al.*, 2009) were applied to the membrane as positive controls. The membrane was baked at 80°C for 40 min, followed by pre-hybridization in DIG Easy Hyb Hybridization buffer (Roche Diagnostics) for 1 h at the optimal hybridization temperature, and hybridization with DIG-labelled probes at the same temperature for 2 h. It was then washed twice for 5 min in Low Stringency Wash Buffer (2× SSC + 0.1% SDS) at room temperature followed by two 15 min washes in High Stringency Wash Buffer (0.1× SSC + 0.1% SDS) at the optimal post-hybridization temperature. The membrane was further washed for 5 min in Washing Buffer (1× maleic acid buffer + 0.5% Tween 20, Roche Diagnostics), blocked for 30 min with 1% Blocking Solution (Roche Diagnostics) in maleic acid buffer, and then incubated for 30 min in Antibody solution (3 µl Anti-DIG-Alkaline Phosphatase conjugate, Roche Diagnostics, in 15 ml Blocking Solution). After washing twice in Washing Buffer and equilibration of the membrane with Detection buffer [0.1 M Tris-HCl (pH 9.5) + 0.1 M NaCl, Roche Diagnostics] for 5 min, DIG/anti-DIG conjugates were detected using 80 µl nitroblue tetrazolium chloride/5-bromo-4-chloro-3-indolyl-phosphate solution (Roche Diagnostics) in 4 ml Detection Buffer.

The *Synergistetes*-specific probes 3.3_65, A_487 and A_845 (Vartoukian *et al.*, 2009) were synthesized with a 3′ DIG label. Optimal conditions for colony hybridization were determined by performing hybridization with a panel of reference strains, clones and amplicons at hybridization and post-hybridization temperatures ranging from 36–48°C and 41–58°C respectively. Cluster A *Synergistetes* clones used for probe validation were: A2G_1 (OTU 1), A3G_42 (OTU 2), A2G_84 (OTU 3.1), A3A_4 (OTU 3.2), A3G_46 (OTU 3.3), A3A_48 (OTU 4.1), A3A_60 (OTU 4.2), A3G_41 (OTU 4.4), A3G_7 (OTU 5), A2G_67 (OTU 6.1) and A3E_52 (OTU 6.2) (Vartoukian *et al.*, 2009). Strains used were *Pyramidobacter piscolens* DSM 21147^T^, *Jonquetella anthropi* E3-33, *Selenomonas sputigena* DSM 20758^T^, *P. baroniae* DSM 16972^T^, *Prevotella oralis* NCTC 11459^T^, *Prevotella intermedia* DSM 20706^T^, *Slackia exigua* ATCC 700122^T^, *Aggregatibacter actinomycetemcomitans* ATCC 33384^T^ and *Fusobacterium nucleatum* ssp. *nucleatum* ATCC 25586. Positive and negative control probes (EUB338 and NON338) were included in all validation experiments. Probes were used at a concentration of 20 pmol ml^−1^ in DIG Easy Hyb hybridization buffer (Roche Diagnostics). Strains were transferred from anaerobic cultures on Fastidious Anaerobe Agar (FAA, Laboratory M) supplemented with 5% horse blood, to nylon membranes. Clones were tested as: (i) whole colonies transferred onto membranes from overnight cultures on LB + 50 µg ml^−1^ kanamycin; and (ii) amplified clone inserts prepared by ‘touch’ PCR using M13 primers as described previously (Vartoukian *et al.*, 2009), and applied to membranes after heat denaturation. In the case of the whole-cell clones, the colony hybridization protocol was modified with an initial DNA denaturation step involving: (i) placement of the colony-transferred-membrane onto filter paper soaked in Denaturation solution (0.5 M NaOH, 1.5 M NaCl) for 15 min; (ii) transfer to filter paper soaked in Neutralization solution (1.5 M NaCl, 1.0 M Tris-HCl, pH 7.4) for 15 min; and (iii) a final 5 min incubation on filter paper soaked in 2x SSC.

Probes A_487, A_845 and 3.3_65 were ultimately used on mixed cultures from clinical samples at hybridization temperatures of 36.5°C, 36.5°C and 46.0°C, respectively, and post-hybridization temperatures 5°C greater than each corresponding hybridization temperature, under which conditions, probes A_487 and A_845 targeted Cluster A *Synergistetes*, and probe 3.3_65 targeted Cluster A *Synergistetes* OTUs 1, 2, 3.1, 3.3, 6.1 and 6.2.

Subgingival plaque samples from subjects F7 and F9 were tested by FISH for the presence of Cluster A *Synergistetes*. Within 30 min of sample collection, triplicate plates of pre-reduced FAA/5% horse blood (subject F7) or BA/5% horse blood (subject F9) were inoculated with 50 µl each of 10^−3^–10^−6^ dilutions of the sample. Plates were incubated in an anaerobic workstation at 37°C. After 5 days of incubation, cells were harvested from one quarter of the plate, suspended in PBS and subjected to FISH with *Synergistetes*-specific probes. *Synergistetes*-positive plates were replica-plated at 7 days using sterile velveteen squares (Scienceware). Colony hybridization was performed on blots lifted from primary or replica plates after 7 or 14 days of incubation. Colonies located on the plate cultures that corresponded to hybridization detections targeted by multiple *Synergistetes* probes were streaked onto fresh plates, subcultured until pure and identified by 16S rRNA gene sequence analysis. In addition, the harvested cells were diluted and used to inoculate fresh BA plates, which were subjected to colony hybridization. Some passage plates were cross-streaked with *Staphylococcus aureus* NCTC 6571 and strains isolated from the subgingival plaque samples.
